# DENGUE VIRAL INFECTIONS

**DOI:** 10.4103/0019-5154.60357

**Published:** 2010

**Authors:** Padmalal Gurugama, Pankaj Garg, Jennifer Perera, Ananda Wijewickrama, Suranjith L Seneviratne

**Affiliations:** *From the Colombo South Teaching Hospital, Sri Lanka*; 1 *From the Children's Hospital at Westmead, Westmead, NSW, Australia*; 2 *From the Department of Microbiology, University of Colombo, Colombo, Sri Lanka*; 3 *From the Department of Infectious Diseases Hospital, Colombo, Sri Lanka*; 4 *From the Department of Clinical Immunology, St. Mary's Hospital and Imperial College, London, UK.*

**Keywords:** *Dengue*, *epidemiology*, *clinical features*, *treatment*

## Abstract

Dengue viral infections are one of the most important mosquito-borne diseases in the world. Presently dengue is endemic in 112 countries in the world. It has been estimated that almost 100 million cases of dengue fever and half a million cases of dengue hemorrhagic fever (DHF) occur worldwide. An increasing proportion of DHF is in children less than 15 years of age, especially in South East and South Asia. The unique structure of the dengue virus and the pathophysiologic responses of the host, different serotypes, and favorable conditions for vector breeding have led to the virulence and spread of the infections. The manifestations of dengue infections are protean from being asymptomatic to undifferentiated fever, severe dengue infections, and unusual complications. Early recognition and prompt initiation of appropriate supportive treatment are often delayed resulting in unnecessarily high morbidity and mortality. Attempts are underway for the development of a vaccine for preventing the burden of this neglected disease. This review outlines the epidemiology, clinical features, pathophysiologic mechanisms, management, and control of dengue infections.

## Introduction

The dengue virus and their mosquito vectors had a wide distribution across many tropical countries in three continents for more than 200 years. In the earlier phase, there were long intervals (10-40 years) between reported dengue epidemics because of minimal chances of exposure (limited intercontinental travel) of susceptible populations to new serotypes. Also, transport of dengue viruses and their mosquito vector across sea borders was slow, impairing their ability to survive.

A pandemic of dengue spread in Southeast Asia with its epicenter in Manila in 1954. By 1975, dengue infections became a frequent cause of morbidity and mortality particularly among the children in countries of South East Asia. In the last decade, epidemics caused by multiple serotypes (hyperendemicity) have become increasingly frequent. Striking changes in the pattern of disease occurrence has led dengue to become one of the most important mosquito-borne viral diseases in the world.

Significant outbreaks now occur in five of the six World Health Organization (WHO) regions.[1,2] The populations at risk of acquiring dengue viral infections include those in urban tropical and subtropical areas and constitutes 40% of the world population.[2] Annually, 100 million cases of dengue fever and half a million cases of dengue hemorrhagic fever (DHF) occur globally, with an average case fatality rate of around 5%.[2,3] Up to 90% of patients with DHF are children less than 15 years of age.[2] There has been a shift in the serotypes causing major dengue epidemics.[4] In Asian regions, the predominant dengue serotype of DEN-2 has been replaced with DEN-3.[5,6]

In 1998, a major pandemic of dengue viral infections occurred in 56 countries, with 1.2 million cases of dengue fever and DHF being reported. A new DEN-3 virus subtype (subtype III) emerged and expanded from the Indian subcontinent.[7] A similar pandemic also occurred in 2001-2002.

Reasons for the global resurgence and spread of dengue fever and DHF epidemics are not entirely clear.[4] Potential reasons are population growth, uncontrolled urbanization in tropical and subtropical countries, proliferation of breeding sites for *Aedes* mosquitoes and the lack of effective mosquito control. These factors increase the distribution and density of the vectors.[8] The rapid evolution of dengue viruses (genotypes associated with increased virulence has expanded from South and Southeast Asia into Pacific region and the Americas) is also responsible. Also, more virulent genotypes of the virus have replaced the less virulent ones.[9]

## Epidemiological Trends

### South Asia

In India, dengue has been endemic for over two centuries with mostly a benign and self limited course. However, similar to the trends in other regions, dengue infections are manifesting in severe forms of DHF and dengue shock syndrome (DSS). Also, there is an increasing frequency of outbreaks. The first major outbreak of dengue in India was reported in 1991. The first epidemic of DHF occurred in Delhi 5 years later.[10] DENV2 was the main serotype involved in 1996 epidemic.[11] However, all four serotypes of dengue viruses were found in co-circulation during the epidemic in 2003.[12] The demographic picture of serologically confirmed cases has remained unchanged since last decade. The predominant age group affected has been young adults (21-30 years), and most cases were reported in the post-monsoon season.

In 2006, another major epidemic affected India and strained the already stretched healthcare system.[13] The average total economic burden was estimated to be US$27.4 million (US$25.7—29.1 million).[14] Dengue-like illness was reported from Sri Lanka in mid 1960s.[15,16] The first major epidemic of DHF occurred in Sri Lanka in 1989-90. Regular epidemics have occurred subsequently resulting in increasing numbers of cases each year. The DEN-3 subtype III and DEN-2 serotype has been identified as major serotypes in Sri Lanka.[7,17] Recent dengue epidemics have also been reported from Pakistan, Bangladesh, and other countries of the region.[18,19]

### The far East

The Republic of China is the most affected country in this region. However, the intensity of dengue fever/DHF epidemics as well as mortality in this region is less when compared with the South East and South Asian regions.[20] The first dengue fever epidemic occurred in China in 1978, and was followed by an epidemic of DHF (DEN-2 serotype) in Hainan Island in 1985-86.[21] In 2002, Hong Kong identified its first locally infected case of dengue infection.[22]

In Australia, dengue and the mosquito vector occurs only in the state of Queensland. Outbreaks occur when the virus is transmitted to the local mosquito population by infected international travelers or overseas residents returning home.[23] Major epidemics of dengue have also occurred in Fiji[24]

### South East Asia

The number of reported dengue cases and incidence of DHF have increased in many countries of South East Asia.[2] The total number of reported cases of DF/DHF from 10 countries of the South East Asian region for the period 1996-2006 is shown in Table 1. All countries except Democratic Peoples' Republic of Korea reported indigenous transmission of DF/DHF. The attack rates and disease patterns are different and dynamic across the region.[25,26] Indonesia has superseded Thailand in having the highest number of dengue cases in the region. DEN-3 has replaced DEN-2 as the predominant serotype during serological surveys.[27] DEN-4 serotype is more common in secondary dengue infections.[28]

**Table 1:** Reported cases of dengue fever/dengue hemorrhagic fever from 10 countries of the South East Asian region for the period 1996-2006 (WHO)

Country	1996	1997	1998	1999	2000	2001	2002	2003	2004	2005	2006
Bangladesh	0	0	0	273	5555	2430	6104	486	3934	1048	2198
Bhutan	0	0	0	0	0	0	0	0	2579	11	116
India	16517	1177	707	944	650	3306	1926	12754	4153	11953	11251
Indonesia	44650	30730	72133	21134	33443	45904	40377	51934	79462	95279	106425
Maldives	0	3	1750	118	180	73	27	38	742	1126	2768
Myanmar	1854	4500	13002	5828	1884	15695	16047	7907	7369	17454	11383
Nepal	0	0	0	0	0	0	0	0	0	0	18
Sri Lanka	1298	980	1275	1688	3343	4304	8931	4749	15408	5686	11906
Thailand	37929	101689	129954	24826	18617	139327	114800	62767	38367	45893	42456
Timor leste	0	0	0	0	0	0	0	0	434	1128	163
Total	102248	139079	218821	54811	63672	211039	188212	140635	152448	179780	188684

Climatic conditions have been considered an important reason for the increasing burden and intensification of dengue virus transmission in the region.[29]

### The Americas

Cuba is the first Latin American country to report a major epidemic of dengue (DEN-1 serotype) fever in 1977-78. The first epidemic of DHF (DEN-2 serotype) was reported in 1981. Cuba achieved a dengue-free period for 16 years using successful vector control program until 1997, when another DEN-2 epidemic affected the region. Children were not affected during this epidemic as they mostly had the primary infections.[30] Venezuela reported first dengue epidemic in 1989.[30]

The incidence of dengue fever has increased significantly in Latin America during the last two decades with more than a million cases reported in 2002 from 30 countries.[31] The present epidemiological trend of dengue in USA is similar to that seen in Asia, with DHF epidemics recurring at 3 to 4 yearly intervals.[31]

### Africa and the middle East

Epidemic dengue fever due to all serotypes have been reported from countries of East Africa as well the Seychelles (1977), Kenya (1982, DEN-2), Mozambique (1985, DEN-3), Djibouti (1991-92, DEN-2), Somalia (1982, 1993, DEN-2), and Saudi Arabia (1994, DEN-2). For unknown reasons, epidemic DHF has not been reported to date in many countries of Africa or the Middle East.[4] It is hypothesized that ethnicity may confer people of African origin an inherent resistance to the disease.

## Characteristics of the Dengue Virus

Dengue is caused by a single-stranded RNA virus belonging to the genus *Flavivirus*. It consists of four closely related serotypes (DEN 1-4) classified according to biological and immunological criteria.[32,33] The mature dengue virion consists of a single-stranded RNA genome surrounded by an icosahedral or isometric nucleocapsid about 30 nm in diameter. This nucleocapsid is covered by a lipid envelope. The complete virion is about 50 nm in diameter and the viral genome is approximately 11 kb in length. The complete or nearly complete sequences of many other flaviviruses, including dengue-1,[34] dengue-2,[35] dengue-3,[36] and dengue-4[37] have been elucidated.

The envelope proteins, with which the virus binds to host cell receptors, carries out biological functions of the virus including transport of the viral genome into the host cell, hemagglutination of erythrocytes, induction of neutralizing antibodies, and protective immune responses.

Seven nonstructural (NS1, NS2a, NS2b, NS3, NS4a, NS4b, and NS5) proteins are involved in the pathogenesis of severe disease. NS1 is involved in viral RNA replication. It gets expressed on the surface of infected cells, without forming part of the virion.[38] Levels of secreted NS1 (sNS1) in plasma positively correlate with viral titers.[39] The higher levels of NS1 in secondary dengue might imply an important role for NS1 in the formation of circulating immune complexes in causing severe dengue.[38]

## Mosquito Vectors in Dengue Infections

Three major species of mosquitoes belonging to the genus *Aedes,* namely, *A. aegypti, A. albopictus,* and *A. polynesiensis* are known to act as vectors in transmission of dengue infections. *A. aegypti* (most important vector) is mainly a container breeder and day biting mosquito, and is found in tropical and subtropical areas.[40] They rest indoors mainly in living rooms and bedrooms, and are active during dusk and dawn. This increases man-vector contact and contributes to difficulty in controlling disease transmission.[41]

*A. albopictus* and *A. polynesiensis* act as vectors depending on the geographic location.[2] *A. albopictus*, originally indigenous to South East Asia, islands of the Western Pacific, and Indian Ocean, has spread to Africa, the Middle-east, Europe, and the Americas. This increases the risk of epidemics of arboviral diseases in these countries. *A. albopictus* also serves as a maintenance vector of dengue in rural areas of dengue-endemic countries of South East Asia and Pacific islands. *A. albopictus* is fortunately not an urban vector of dengue, except in countries where *A. Aegypti* is absent (parts of China, the Seychelles, Japan, and Hawaii).

The mosquito eggs of *Aedes* can survive for long periods, as they are capable of withstanding desiccation. Improper disposal of garbage and inadequate wastewater drainage facilitates are responsible for high mosquito densities. Significant increases in the mosquito larval populations are seen during and after the rainy season. This causes post-monsoon epidemics of dengue in the South and Southeast Asian countries.[42] Warm and humid temperatures shorten the viral replication time within the female mosquitoes.[43,44] After biting an infected human, dengue viruses enter an adult female mosquito and gain access to different insect tissues. Viruses in the salivary glands of the infected mosquitoes are transmitted to another human with next blood meal. Infected mosquitoes take a relatively longer period for a blood meal that enhances the efficiency of *A. aegypti* as a dengue viral vector.[45] Both transovarial and male to female sexual transfer have been documented.[46] This allows the propagation of virus to their progeny during interepidemic periods without human or other vertebral host participation.

## Clinical Manifestations of Dengue Infections

Infection with dengue virus may be subclinical or may cause illness ranging from a mild undifferentiated fever to a severe, life-threatening presentation (DHF or DSS). The symptoms may persist for more than a week and even longer in some patients. The differential diagnosis of dengue fever and DHF is shown in Table 2.

**Table 2:** Differential diagnosis of dengue fever and dengue hemorrhagic fever

Dengue fever
Infectious mononucleosis
Chikungunya viral infections
Coxsackie and other enteroviral infections
Rickettsial infections
Rubella
Parovirus B19 infections
Influenza
Leptospirosis
DHF
Leptospirosis
Chikungunya viral infections
Hanta viral infections
Other hemorrhagic fever
Meningococcal septicemia
Yellow fever
Kawasaki disease

### Undifferentiated fever

This usually follows a primary infection but may also occur during the early phase of a secondary infection. Clinically it mimics many other viral infections and is frequently not diagnosed.

### Dengue fever

Dengue fever is most commonly seen in adults and older children. It occurs during both primary and secondary infections. Typically symptoms start with sudden onset of high fever, which could be biphasic lasting 3 to 7 days.[47,48] Other symptoms include intense headache (especially behind the eyes), fatigue, muscle and joint pain (ankles, knees, and elbows), unpleasant metallic taste in mouth, loss of appetite, vomiting, diarrhea, and abdominal pain. Dermatological manifestations such as flushed skin (on face and neck), a macular papular rash, or a fine skin rash on the arms and legs as the fever subsides are seen in many patients. Severe itching, peeling of skin, and hair loss are also seen. Young patients may present with coryza, diarrhea, rash, seizure, vomiting, headache, and abdominal pain.[49]

Hemorrhagic manifestations are uncommon in dengue fever. However, minor bleeding (nose or gums), heavy menstrual periods, petechiae/purpura, and gastrointestinal bleeding may be seen.[47,50] A positive tourniquet test has been reported in many individuals with dengue fever.[51] Recovery from dengue fever is usually uneventful, but may be prolonged especially in adults.[2]

### Dengue hemorrhagic fever

Dengue hemorrhagic fever (DHF) usually follows a secondary dengue infection. In infants, it may follow a primary infection due to maternally acquired dengue antibodies.[52] Typically, DHF is characterized by high fever, hemorrhagic phenomena, features of circulatory failure, and hepatomegaly.[53] DHF is divided into four grades according to severity [Table 3]. The clinical course of DHF is divided into three phases, namely, febrile, leakage, and convalescent phases. The febrile phase begins with sudden onset fever accompanied by generalized constitutional symptoms and facial flush. The fever is high grade (usually >39°C), intermittent, and associated with rigors.[48] Clinical manifestations similar to DF are seen. A morbilliform rash and bleeding manifestations appear in the early febrile phase.[54] The fever lasts for 2-7 days and then falls to normal or subnormal levels when the patient either recovers or progresses to the plasma leakage phase.[55]

**Table 3:** Grading of dengue hemorrhagic fever

Grade	
I	No shock, only positive tourniquet test
II	No shock, has spontaneous bleeding other than a positive tourniquet test
III	Shock
IV	Profound shock with un-measurable blood pressure and/or pulse

Patients remaining ill despite normalization of temperature progress to DHF. Onset of plasma leakage is characterized by tachycardia and hypotension. The patient sweats, becomes restless, and has cold extremities. In less severe cases, the changes are minimal and transient, reflecting a mild degree of plasma leakage. Most patients recover from this stage spontaneously or after a short period of fluid and electrolyte replacement. In severe cases with high plasma leakage, patients may develop full blown circulatory shock characterized by prolonged capillary refill time and narrow pulse pressures.

During the phase of plasma leakage, pleural effusions (usually right side) and ascites are common.[56] Pericardial effusions may also be seen. Myocarditis is associated with increased morbidity and mortality. Thrombocytopenia and hemoconcentration are usually detectable before the subsidence of fever and the onset of shock.

In DHF, bleeding may occur from any site and does not correlate with the platelet count. Hemorrhagic manifestations occur after fever has settled.[54] The commonest site of hemorrhage is the gastrointestinal tract (which manifests as hematemesis or melena), followed by epistaxis.[57] Vaginal bleeding has been reported in females despite high platelet counts. Bradycardia and a confluent petechial rash with erythema and islands of pallor are seen during convalescence period.

### Dengue shock syndrome

DSS is associated with very high mortality (9.3-47%). Presentation is typical of circulatory failure such as tachycardia, hypotension, cold blotchy skin, congested peripheries, and circumoral cyanosis. It is important to distinguish DSS from septic shock to avoid inappropriate use of antibiotics.[58]

Patients with DSS die due to multiorgan dysfunction and disseminated intravascular coagulation. Most patients remain fully conscious to the terminal stage. The duration of shock is short and the patient rapidly recovers with appropriate supportive therapy. DSS may be accompanied by encephalopathy due to metabolic or electrolyte disturbances. Adequate urine output and the return of appetite are considered to be good prognostic signs.

## Laboratory Findings

Platelet counts and serum biochemistry are normal in most cases of dengue fever. Leucopenia, thrombocytopenia, and raised liver enzymes are seen in a significant proportion of patients. Thrombocytopenia (usually < 100 × 10^9^/l) and hemoconcentration (20% rise from basal level) are constant findings in DHF. The white cell count may vary from leucopenia to mild leukocytosis. Significant leucopenia and neutropenia are seen at the end of the febrile phase.[51] Atypical lymphocytosis (>15%) is commonly seen before defervescence.

A transient mild albuminuria and occult fecal blood are common. In most cases, reduced fibrinogen, prothrombin, Factor's VIII, XII and antithrombin III, and raised fibrinogen degradation products are observed. Impaired platelet functions and low complement levels (C3 in particular) are also documented. APTT and PT are prolonged in upto half of DHF patients.

In severe cases, liver dysfunction with the elevation of alanine and aspartate aminotransferase levels occur, more so in DHF.[51] Electrolyte abnormalities, metabolic acidosis, and increased blood urea are frequently seen during prolonged shock. Lipid levels (HDL and LDL in particular) are diminished in severe forms of DHF.[59] In DHF and DSS, pleural effusions may correlate with disease severity.

## Complications of DHF

The severe complications of dengue infections such as liver failure, disseminated intravascular coagulation, encephalopathy, myocarditis, acute renal failure, and hemolytic uraemic syndrome are rare but have been noted to be more frequent in recent epidemics.[60]

### Liver failure

Although the liver is involved in all types of dengue viral replication, DEN-3 or DEN-4 serotypes produce greater liver involvement.[61,62] One-third of dengue infections experience liver derangements. Histopathological changes such as fatty changes, centrilobular necrosis, and monocyte infiltration of portal tract have been described.

Strong correlation between T-cell activation and hepatic cell infiltration have been noted.[63] Fulminant liver failure due to hepatitis or focal necrosis of the liver and hepatic encephalopathy has been reported.[64]

### Encephalopathy

Dengue infections can cause headache, seizure, depressed sensorium, behavioral disorders, neck stiffness, delirium, paralysis, cranial nerve palsies, and coma.[65]. Encephalopathy in dengue infections have been ascribed to hepatic dysfunction, electrolyte imbalances, cerebral edema, hypoperfusion, cerebral hemorrhage, hyponatremia, cerebral anoxia, microcapillary hemorrhage, release of toxic products, and dengue encephalitis.[66]

### Myocarditis

Acute reversible myocardial dysfunction is the commonest documented cardiac complication. The variable incidences of dengue myocarditis had been postulated to be due to variable immunopathogenesis secondary to variations in serotypes. Dengue myocarditis is generally reversible with favorable outcomes if diagnosed and treated early.[67] Alternation of autonomic tone, rhythm disorders, such as atrioventricular blocks and ventricular ectopic beats, ST segment and T wave changes, low ejection fractions, and global hypokinesia on radionuclide ventriculography have been reported. Although dengue pericarditis has been described as an extension of myocarditis, dengue endocarditis is not known.[68]

## Pathogenesis of Dengue Fever/DHF

Peak plasma viraemia correlates with the severity of dengue infections.[39] The pathophysiological mechanisms can be described due to viral pathogenesis, viral genotypes and virulence, antibody, cellular, cytokine, and innate immune responses to the dengue virus.

### Viral pathogenesis

Infection with one serotype of dengue confers future protective immunity only against that serotype. Infection by a different serotype generally results in a more severe infection due to antibody-dependent enhancement. There might be other infections contributing to disease severity of secondary dengue infections.[32]

Dengue virus replicates within mononuclear cells including skin dendritic cells, tissue macrophages, peripheral blood monocytes, and hepatocytes. Endothelial cells do not appear to be host cells for dengue virus replication.[69] There is currently uncertainty regarding the major host cell receptors involved in viral entry of dengue.

### Dengue viral genotypes and virulence

The dengue viruses display 30% divergence across their polyproteins. Also, genetic variations exist within each serotype resulting in genetically distinct genotypes.[70] At present, 3, 6, 4, and 4 genotypes have been identified for the four dengue virus serotypes. Multiple genotypes often cocirculate within the same geographic area. DEN-2 and DEN-3 serotypes with Asian origins have been established as endemic cycles in other continents. These genotypes are known to produce higher viral titers.[71] The evolution of dengue viruses during an epidemic and its association with epidemic severity are researchable issues. The different genotypes involved in primary and secondary infections attribute to different severity of the clinical illness.[72]

### Antibody responses and dengue viral infections

During secondary dengue infections, pre-existing antibodies form complexes with the dengue virus. The Fc portion of these antibodies binds to cells bearing Fc RI and Fc RII receptors causing infection of large numbers of cells by the dengue virus.[73] Anti-NS1 antibodies induce endothelial cell apoptosis. Total and dengue-specific IgE antibody levels are elevated in DHF and DSS than dengue fever.

Thrombocytopenia in DHF is caused by antiplatelet antibodies (IgM) and platelet cross-reactive dengue viral antibodies, defective platelet production, and peripheral destruction in liver and spleen. IgM and IgG antibodies against dengue virus provide immune protection by several mechanisms such as blocking cellular attachment, viral fusion, or antibody-dependent cellular cytotoxicity (ADCC). Dengue viral epitopes targeted by neutralizing antibodies have been identified.[74]

The overall incidence of severe infections is relatively low compared to the large number of dengue cases reported annually. This may be due to the cross-reactive dengue-neutralizing antibodies blocking productive infection.[75] It has been suggested that the longer the gap between primary and secondary infections, the more severe are the clinical manifestations.

### Cellular immune responses in dengue infections

The dengue virus can infect both CD4+ and CD8+ T-cells.[76] The serotype cross-reactive memory T-cells formed following primary infection augment infection by producing various cytokines during secondary infection. Dengue-infected CD4+ T-cells produce a unique cytokine called cytotoxic factor (CF), which increase capillary permeability, cause cerebral edema, and change blood leukocytes.

Dengue virus infected CD4+ T-cells also produce IFN-γ, TNF-α, and TNF-β, which contribute to the pathogenesis of secondary dengue. Dengue infections are associated with decreased numbers of CD4+ T-cells, CD8+ T-cells, and natural killer (NK) cells. Reversal of CD4:CD8 ratios tends to occur during convalescence. Activated CD4+ T-cell clones are capable of destroying nonantigen presenting target cells such as hepatocytes.

Suppression of T-cell responses 2 weeks after the onset of dengue fever predisposes to respiratory tract infections or diarrhea after dengue infections.[56] IL-10, whose levels are increased in DHF, is known to downregulate antigen-presenting cell responses and induce unresponsiveness in T-cells. In DHF, B-cell numbers are increased while T-cells may be reduced partly due to massive activation, proliferation, and programmed cell death of dengue-specific T-cells.

### Cytokine responses in dengue infections

Monocytes, B-cells, and mast cells infected with the dengue virus produce different cytokines. The types and levels of cytokines vary during the course of illness. Tumor necrosis factor-α (TNF-α), interleukin (IL)-2, IL-6, and IFN-γ are highest in the first 3 days of illness, whereas IL-10, IL-5, and IL-4 appear later.[77] Th1 responses seen during the first 3 days are replaced by Th2 responses in the later phase of illness. Il-8 and IL-12 have opposite roles in the clearance of virus and host recovery as IL-12 becomes undetectable in severe infections, whereas serum IL-8 is elevated. IL-8 exerts potent proinflammatory and chemo-attractant activity causing enhanced disease severity. Other cytokines correlating with severe DHF are elevated serum level of IL-6, IL-13, and IL-18.

### Innate immunity and dengue viral infections

Both type I and type II interferons have been shown to control viral replication *in vitro*. In acute dengue, activated NK cells may be involved in killing infected cells by ADCC or cytokine release. Dengue virus infected dendritic cells secrete proinflammatory and immune-modulating cytokines. In addition, in acute dengue, soluble NS1 activates the complement system.

### Host genetic influences in dengue viral infections

The diversity of clinical manifestations in dengue infections raises a possibility of a genetic basis to the regulation of disease expression. Resistance to DHF observed in Cubans of African descent and lower (2%) and higher (30%) incidences of DHF in Negroid and Caucasian populations supports a genetic hypothesis. Some disease associations have been shown in human leukocyte antigen (HLA) alleles, the vitamin D receptor, and FcγIIa. Polymorphism in HLA class 1 loci was found to be associated with increased susceptibility to DHF in Vietnam.[78] This association was confined to the HLA-A region and not the *HLA-B* gene. Children with HLA-A*24 were more susceptible for DHF compared to children with HLA-A*33. In addition, HLA-A*0203 was found to be associated with less severe dengue infections irrespective of the infecting virus serotype in secondary infections. Polymorphism in five non-HLA genes namely, *IL-4*, *IL-1RA*, *MBL*, *VDR*, and *FccRII* were found to increase susceptibility to DHF. A functional mutation in the promoter region of DC-SIGN was associated with susceptibility to mild dengue, but not DHF.[79] The immune-regulatory action of vitamin D receptor includes monocytes activation, stimulating cellular immune responses, and suppressing immunoglobulin production and lymphocyte proliferation.

## Laboratory Diagnosis of Dengue Infections

Specific methods used for the diagnosis of dengue infections include virus isolation, serology, and molecular techniques [reverse transcriptase-polymerase chain reaction (RT-PCR)]. Laboratory diagnosis of dengue must consider the timing of clinical course and relevant parameters in their quantitative patterns. Each method has its advantages and limitations.

### Virus isolation and identification

Virus isolation is a gold standard for diagnosing DENV infections. Serotypes of DENV are determined using immunofluorescence (IF) staining in infected cells with serotype-specific monoclonal antibody. Viral isolation is useful when the samples are collected in early phase of disease (within 6 days). Dengue viruses can be isolated from serum, plasma, or leucocytes during the febrile phase and also from postmortem specimens such as liver, lung, spleen, lymph nodes, thymus, cerebrospinal fluid, or pleural/ascitic fluid. In secondary infection, neutralizing antibodies interfere with virus isolation.

The most sensitive virus isolation method is *in vivo* amplification through mosquito inoculation. Mosquito-derived cell cultures such as C6/36 (*Aedes albopictus*), AP61 (*A. pseudoscutellaris*), and TRA284 (*Toxorhynchites amboinensis*) are used in clinical laboratories. The IF assay is preferred in clinical practice as it is cheaper and provides results faster (24-48 hours).[80]

### Serological diagnosis

Serological techniques include hemagglutination inhibition tests, enzyme-linked immunosorbent assay (ELISA), complement fixation test, and neutralization tests. Dengue IgM and IgG ELISA are sensitive (83.9-98.4%) and specific (100%), less expensive, quick, and simple tests to perform. Dengue IgM antibodies appear in serum by the fifth day of infection and become undetectable by 30-60 days of illness. The conventional or capture ELISAs have been used to identify different dengue viral serotypes.[81] A fourfold or greater rise in antibody titers in paired samples is better than single random sampling.

### Molecular detection

RT-PCR is a valuable diagnostic tool with high sensitivity and specificity even before dengue-specific antibodies are produced. RT-PCR is more sensitive when compared to virus isolation and also identifies the circulating serotype.[82] It also avoids interference due to cross-reactivity of dengue serotypes with other flaviviruses. The disadvantage is its high cost and the expertise needed.

## Risk Factors for the Development of DHF

Risk factors for the development of DHF include serotype and virulence of the infecting virus, age, sex, immune status, and genetic background of the host.[32] The highest case fatality and hospitalization rates due to DHF/shock occur in infants, elderly, people suffering from other chronic illnesses, and females.[83] The DEN-2 virus replicates better within peripheral blood mononuclear cells from asthmatics. Malnutrition is uncommon among patients with DHF, compared with patients with other infectious diseases.[84]

## Management of Dengue Infections

Management of dengue viral infections is primarily symptomatic.

### Management of dengue fever

Fever is treated with paracetamol. Nonsteroidal anti-inflammatory drugs such as aspirin or ibuprofen should be avoided. Tepid sponging is helpful. An antiemetic such as domperidone and a proton pump inhibitor (pantoprazole) could be used. In the early phase of illness, increased oral fluids are given; intravenous fluids should be used in the presence of severe vomiting or dehydration.

Monitoring platelet counts and packed cell volumes should be done daily from the third day of fever and continued through the leakage phase until recovery. The indications for hospitalization in dengue include poor oral intake to prevent dehydration and complications such as bleeding, changes in the level of consciousness, or laboratory evidence of DHF.

### Management of DHF

DHF is divided into four grades of severity based on clinical manifestations. The cornerstone of management is close surveillance of fluid and electrolyte balance. The packed cell volume should be monitored 4-6 hourly. A platelet count <100,000 and a >20% rise in packed cell volume reflect significant plasma loss, mandating prompt, and aggressive volume replacement. The rate of fluid administration is guided by changes in urine output and packed cell volume. The infusion rate should be reduced once the leakage phase ends (12-48 hours). Judicious fluid administration is necessary to avoid respiratory distress secondary to massive pleural effusions/ascites or pulmonary edema. Intravenous fluid therapy could be stopped when the packed cell volume falls to 40% generally heralding the convalescent phase.

The choice of fluids in severe dengue infections has remained a contentious issue. The WHO recommends using crystalloids for volume replacement in DHF. Initial resuscitation using colloids have been shown to restore the cardiac index and pulse pressure and normalize the packed cell volumes sooner than crystalloid solutions.[84]

Significant hemorrhagic manifestations need platelet transfusions. The value of prophylactic platelet transfusions have been questioned for severe thrombocytopenia in uncomplicated dengue.[85] Liver functions should be monitored and an ultrasound scan of abdomen may be needed. Detection of dengue myocarditis is helped by doing an electrocardiogram, cardiac troponin, and a chest radiograph.

### Management of dengue shock syndrome

Dengue shock is a medical emergency. Prompt administration of intravenous fluid to expand plasma volume is essential. Close surveillance of vital parameters is needed. Oxygen saturations are monitored using a pulse oxymeter and investigations including grouping and cross-match, full blood count, renal, and liver function tests should be done. Electrolyte abnormalities, hypoglycemia, and metabolic acidosis should be corrected. Laboratory indicators for the disseminated intravascular coagulation should be done. Fresh frozen plasma, platelet concentrates, or cryoprecipitate are individualized depending upon clinical and laboratory parameters. Two to three intravenous fluid boluses with Ringer's acetate or normal saline may be needed.[86] Dextran 70 and Haesteril are the preferred colloid solutions.[87]

### Monitoring antishock therapy

Standard hemodynamic and clinical variables including pulse, blood pressure, respiratory rate, temperature, hematocrit (every 2 hours during the first 6 hours, and then every 4 hours), fluid balance, and urine output have to be monitored.

### Management of unusual manifestations/complications

Drainage procedures for plural effusions and ascites should be avoided. Patients who develop massive pleural effusions or ascites require prolonged hospitalization for observation. Acute liver and renal failures are frequently encountered unusual manifestations that require standard treatment. No specific treatment is available for encephalopathy. Convulsions and/or coma are managed conservatively. Myocarditis should be suspected if shock persists and should be treated with inotropes and oxygen therapy in an intensive care setting.

### Criteria for discharging patients

Hospitalized patients with DHF/dengue shock syndrome should meet the following criteria before discharge:

- Apyrexia for 24 hours without the use of antipyretics and a return of appetite.
- Objective improvement in clinical picture.
- Stable hematocrit.
- Three days after recovery from shock.
- Platelet count greater than 50,000/mm^3^.
- No respiratory distress from pleural effusion/ascites.

## Prevention and Control of DHF

The prevention and control of dengue infections are currently based mainly on preventing man-vector contact as there is no effective available vaccine. Strategies adopted include environmental, biological, and chemical control, and active case surveillance. Successful control programs warrant incorporation of all methods and a partnership between different dengue control agencies and the community. The dengue control programs in the South East Asia and South Asian regions have been unsuccessful because they have relied solely on insecticide spraying.[2]

### Environmental control methods

Environmental control methods aim at reducing vector breeding sites, solid waste management, modification of man-made breeding sites, and improvements in house design. Public education programs are effective.[88] Personal protection using household insecticidal products or mosquito repellents are of limited utility in dengue control programs as the vector is chiefly a day-biting mosquito.

### Biological control of the vector

Biological control methods are targeted against the larval stages of the dengue vector. They include the use of larvivorous fish such as *Gambusia affinis* and *Poecilia reticulate*, endotoxin-producing bacteria (*Bacillus thuringiensis* serotype H-14 and *B. sphaericus* are currently used), and copepod crustaceans. *B. thuringiensis* serotype H-14 is more effective against *A. aegypti* with very low levels of mammalian toxicity, and has been accepted for use in household containers storing water.[2] High cost of these methods have restricted these efforts to small-scale field operations.

### Chemical control

This includes the application of larvicidal insecticides or space spraying. Space spraying is more widely used as larvicidal insecticides cost more. Insecticides used for treating containers holding water include temephos 1%, sand granules, and insect growth regulators. Regular monitoring of resistance to insecticides is essential.[2] Thermal fogging is the preferred method for space spraying.

### Current status of the dengue vaccine

Research efforts are underway to develop a dengue vaccine that is safe and immunogenic against all four serotypes. Currently there is no vaccine or antiviral drug against dengue viral infections. Cross-protection between dengue virus serotypes is limited. The major challenge is to induce a broad durable immune response against all four serotypes of dengue virus simultaneously while avoiding the possible exacerbation of risk of developing the severe forms of disease through incomplete or modified responses. Recent efforts are to utilize recombinant DNA technology such as synthetic consensus (SynCon) human codon optimized DNA vaccine.[89,90]

## Acronyms and Definitions

ADE: Antibody-dependent enhancement

DENV: Dengue virus

DF: Dengue fever

DHF/DSS: Dengue hemorrhagic fever/dengue shock syndrome

Extrinsic incubation period: The latent period in a vector mosquito before the virus has disseminated to the salivary glands, from where it can be transmitted to a vertebrate host as the mosquito takes a blood meal

Herd immunity: The threshold level of collective immunity in a population, above which transmission of a particular pathogen will be disrupted and not be maintained

HLA: Human leukocyte antigen

House index (HI): The percentage of houses with containers infested with mosquito larvae and/or pupae

Hyperendemic: The presence of numerous serotypes of dengue virus cocirculating in one location

Neutralizing antibodies: Antibodies capable of preventing infection of a cell/host by a pathogen

R_0_: Basic reproductive rate

Secondary infection: A subsequent infection with a heterotypic serotype of DENV, occurring months to years after the primary infection

UTR: Untranslated region

Vector competence: The capacity of a vector to transmit a pathogen by virtue of being susceptible to infection and dissemination and subsequently capable of transmission to an appropriate host

Vector control program (vertical versus horizontal): A vertical control program is a top-down approach in its design and implementation and is usually government led; whereas a horizontal or community-led (bottom- up) program may have institutional support, but focuses primarily on including community participation

Vertical transmission: Transfer of DENV from an infected female mosquito to her offspring, either by transovarial transmission or by infection of the egg at time of oviposition
